# COST-BENEFIT ANALYSIS OF HEARING CARE SERVICES AMONG MEDICARE BENEFICIARIES WITH HEARING AIDS

**DOI:** 10.1093/geroni/igz038.168

**Published:** 2019-11-08

**Authors:** Amber Willink, Amber Willink, Nicholas S Reed, Frank R Lin

**Affiliations:** Johns Hopkins University, Baltimore, Maryland, United States

## Abstract

Hearing care services for older adults with hearing aids are underutilized and are not covered by the Medicare program. Little information exists to the value of hearing care services for older adults with hearing aids. Using the Medicare Current Beneficiary Survey 2013, we conducted a cross-sectional analysis of the impact of hearing care services use on Medicare spending among those with hearing aids. Older Medicare beneficiaries with hearing aids that received hearing care services in the previous 12 months were propensity score matched to those who did not receive services. Average annual Medicare spending was $8196 (CI:$6670-$9723) among Medicare beneficiaries who used hearing care services and $10,709 (CI:$8878-12541) among matched controls. Spending differences were driven by higher skilled nursing facility and home health spending among matched controls. Increasing access to hearing care services among Medicare beneficiaries with hearing aids may provide value to the health care system and Medicare program.

